# The *CFTR* gene variants in Japanese children with idiopathic pancreatitis

**DOI:** 10.1038/s41439-019-0049-7

**Published:** 2019-04-11

**Authors:** Manami Iso, Mitsuyoshi Suzuki, Kumiko Yanagi, Kei Minowa, Yumiko Sakurai, Satoshi Nakano, Kazuhito Satou, Toshiaki Shimizu, Tadashi Kaname

**Affiliations:** 1 Department of Pediatrics and Adolescent Medicine, Juntendo University Graduate School of Medicine, 2-1-1 Hongo, Bunkyo-ku, Tokyo, 113-8421 Japan; 2 Department of Genome Medicine, National Center for Child Health and Development, 2-10-1 Okura, Setagaya-ku, Tokyo, 157-8535 Japan

**Keywords:** Medical genetics, Genotype

## Abstract

The cystic fibrosis transmembrane conductance regulator (*CFTR*) gene has been reported as one of the pancreatitis susceptibility genes. Although many variants of *CFTR* have been reported in Caucasian patients, there are few data in Japanese patients. We aimed to survey *CFTR* variants in Japanese children with idiopathic pancreatitis. Twenty-eight Japanese paediatric patients with idiopathic pancreatitis were enroled, who were not previously diagnosed by genetic analysis of *PRSS1* and *SPINK1*. The entire *CFTR* gene was sequenced in the patients by combining LA-PCR and next-generation sequencing analysis. To determine a splice-affecting variant, *CFTR* expression was investigated in the nasal epithelial cells by RT-PCR. One (3.6%) and 15 (53.6%) of 28 patients had pathogenic and functionally affected variants in the *CFTR* gene, respectively. Two variants, p.Arg352Gln and p.Arg1453Trp, were found more frequently in the patients compared with one in Japanese healthy controls (*p* = 0.0078 and 0.044, respectively). We confirmed skipping of exon 10 in the nasal epithelial cells in one patient having a splice-affecting variant (c.1210-12 T(5)) in intron 9. Functionally affected variants of the *CFTR* gene are not so rare in Japanese paediatric patients with idiopathic pancreatitis. Surveying *CFTR* gene variants in a Japanese sample could help identify pancreatitis risk in these children.

## Pancreatitis: Mutation helps identify risk in Japanese children

Mutations in a cystic fibrosis-related gene could help identify Japanese children at risk of developing pancreatic inflammation. Tadashi Kaname, of Tokyo’s National Center for Child Health and Development, and colleagues sequenced the cystic fibrosis transmembrane conductance regulator gene (*CFTR*) in 28 Japanese children with pancreatitis of unknown origin. The gene is involved in the development of cystic fibrosis and has been reported to be associated with pancreatitis but little is known about its role in idiopathic pancreatitis in Asian populations. The team found *CFTR* gene mutations in 16 out of the 28 children. Cystic fibrosis is uncommon among Japanese, so *CFTR* mutations were also thought to be rare. The study suggests, however, that mutations might not be so rare in Japanese children with idiopathic pancreatitis and could help identify those at risk of developing the condition.

## Introduction

There are many risk factors contributing to acute pancreatitis in children. Genetic analysis can be helpful in making the diagnosis of paediatric pancreatitis. The first report of a genetic cause of idiopathic pancreatitis demonstrated mutations in cationic trypsinogen (*PRSS1*)^1^. Since then, another causative gene for pancreatitis, serine protease inhibitor *Kazal* type 1 (*SPINK1*)^2^, and strongly associated genes, such as, chymotrypsinogen (*CTRC*)^3^ and recently carboxypeptiase A1 (*CPA1*)^4^, have also been reported.

The cystic fibrosis transmembrane conductance regulator (*CFTR*) gene has been identified as a causative gene for cystic fibrosis (CF)^5^ and is also reported to be a gene associated with pancreatitis^6,7^. *CFTR* encodes a protein of 1480 amino acid residues expressed in the apical membrane of exocrine epithelial cells and plays a role as a cAMP-dependent chloride channel^5^. To date, more than 2000 variants of the *CFTR* gene have been reported^8,9^.

Influences of *CFTR* function depend on its variants, which varies with symptoms or appearance of the disease. Many cohort studies examining the association between *CFTR* variants and pancreatitis have been conducted in western countries^6,7,10,11^. However, there are few reports on association between *CFTR* variants and idiopathic pancreatitis in Asian populations, except for a few studying alcoholic chronic pancreatitis^12–16^. Furthermore, there is no information about the genetic risk of *CFTR* variants in Japanese children with idiopathic pancreatitis. In our previous study, we performed genetic analysis of *PRSS1*, *SPINK1*, *CTRC* and *CPA1*, finding that 39% (50/128) of paediatric Japanese patients with idiopathic pancreatitis had at least one pathogenic variant of those genes^17^. However, there is no such data for the *CFTR* gene in this sample. Therefore, the aims of our study were to survey *CFTR* variants in Japanese children with idiopathic pancreatitis to determine any relationship between them.

## Methods

### Subject and data

This study was approved by the ethical review committee of Juntendo University (approval number 2017176) and National Center for Child Health and Development (approval number 1800). Written informed consent was obtained from each subject or their relatives before the study in accordance with the principles of the Declaration of Helsinki.

In total, 28 Japanese paediatric patients with idiopathic pancreatitis were analysed, who had no pathogenic variants of *PRSS1* and *SPINK1* by genetic analysis in a previous study^17^. In addition, their families and 92 healthy Japanese girls (9–12 years old) were enroled as healthy control subjects. In-house data of whole exome sequencing from randomly picked 1500 individuals, who were not related to pancreatitis, were also used as control.

### Targeted next-generation sequencing

Genomic DNA was extracted from peripheral blood leucocytes. Sixteen primer pairs were designed to amplify the entire region of the *CFTR* gene (GenBank: NM_000492), spanning ~200 kb, including the promoter region, which is 2 kb upstream of the translation initiation codon of exon 1 (Supplementary Table S1). Long-range and accurate PCR (LA-PCR) was performed using KOD Multi & Epi (TOYOBO, Co., Ltd, Osaka, Japan) under appropriate conditions listed in Supplementary Table S1. Each LA-PCR product was confirmed by agarose gel electrophoresis and the concentrations were measured on a Qubit fluorometer using dsDNA Broad-Range assay kit (Thermo Fisher Scientific, Waltham, MA, USA). Then, the equal amount of amplicons were mixed and sheared to about 400 bp fragments using Covaris S220 (Covaris, Inc., Woburn, MA, USA) in accordance to the manufacturer’s instructions. The sheared products were then purified using Mini-Elute PCR Purification Kit (Qiagen, Hilden, Germany) and constructed libraries for GS Junior sequencer (Roche, Basel, Switzerland) with MID adaptors using GS Junior titanium Rapid library (shotgun) and emPCR (Lib-L) kits (Roche, Basel, Switzerland). Short fragments were removed using the AMPure beads kit (Agencourt, Beckman Coulter Genomics, Pasadena, CA, USA). Quality and product size were assessed on the 2000 TapeStation (Agilent Technologies, Inc., Santa Clara, CA, USA). Library quantification was determined by fluorometric measurements using a QuantiFluor™-ST Fluorometer (Promega, Inc., Madison, WI, USA). Then, three libraries were mixed at equal quantity and amplified using the Lib-L emPCR Kit (Roche, Basel, Switzerland), following the manufacturer’s instructions. Bead enrichment and sequencing were performed using GS Junior Titanium Sequencing Kit (Roche, Basel, Switzerland).

Sequence data processing, mapping and variant calling were assessed on the built-in software, GS Run Browser and GS Reference Mapper (Roche, Basel, Switzerland). The genomic data of GRCh37 and SNP135 were used as reference for variant calling. In addition, the reads were confirmed visually using the Integrative Genomics Viewer software^18^ and all variants in the exons and some in the introns were confirmed by Sanger sequencing.

### Sanger sequencing

In the next-generation sequencing (NGS) data, the detected variants and low coverage (< 8 reads) of coding regions were validated by the Sanger sequence. Variants that were found in the patients were also sequenced in their families by Sanger sequencing as well; some family members were affected with pancreatitis. We also analysed sequencing data of splicing variants in intron 6 (GATT repeats) and 9 (poly T and TG repeats) from healthy Japanese girls as controls, as we had no information about the variant frequencies of these regions among the Japanese. Primers for the Sanger sequencing are listed in Supplementary Table S2. Sanger sequencing was performed using an ABI3130xl DNA Analyzer (Applied Biosystems, Foster, CA, USA).

### RNA analysis

The nasal epithelial cells were collected by gently brushing the inferior turbinate using a cotton swab^19^. The swab was immediately immersed in a 350 μl buffer RLT preservation solution (Qiagen, Hilden, Germany) and stored at −20 °C. Total RNA was extracted using RNeasy Micro kit (Qiagen, Hilden, Germany) and reverse transcribed to cDNA using PrimeScript™ RT reagent kit (Takara-Bio, Otsu, Japan).

**Fig. 1:**
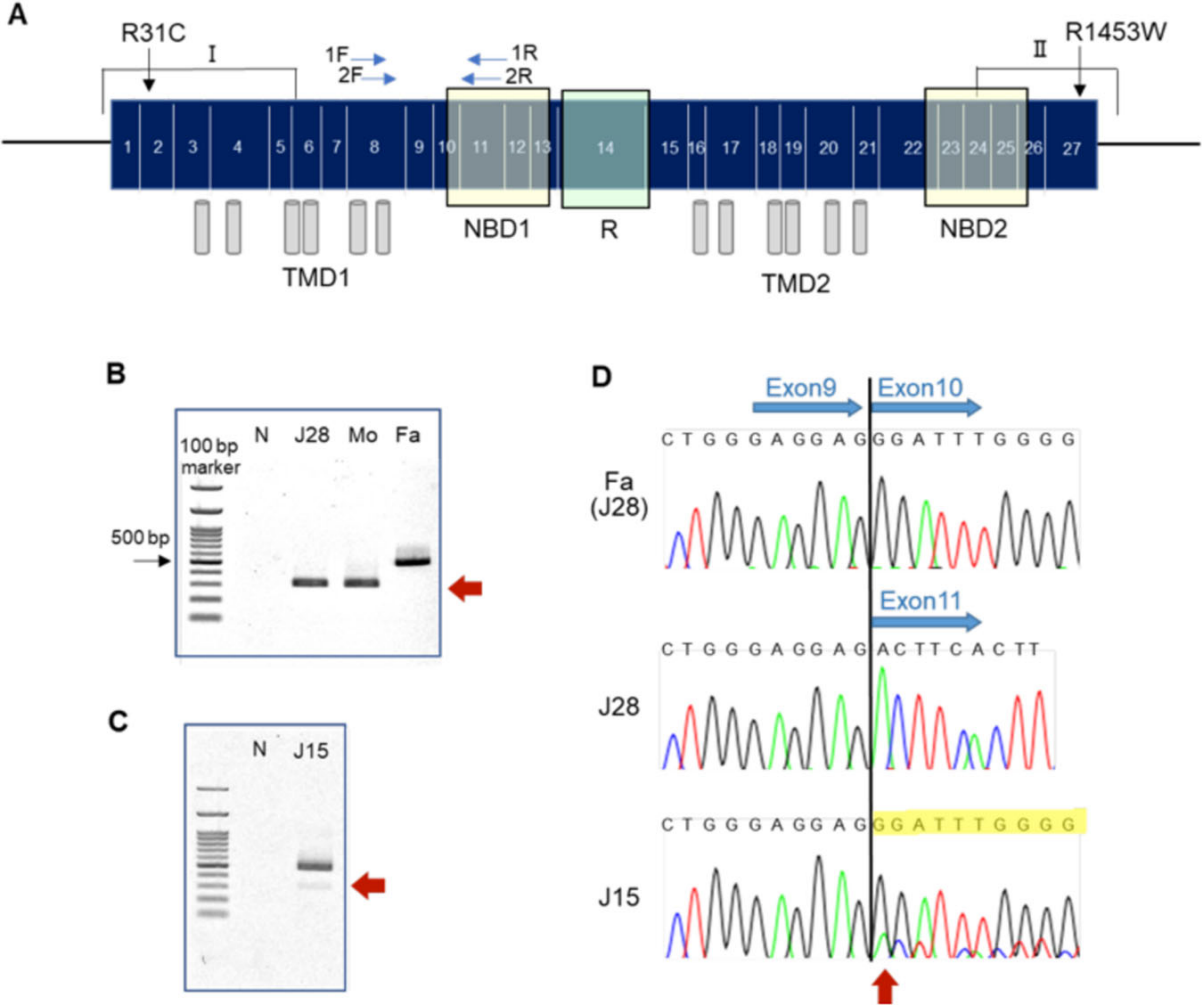
Schematic representation of the CFTR cDNA and primer sites for RT-PCR, and skipping of exon 10. **a** Schematic diagram of the CFTR cDNA (exons) and domains, and amplified regions by RT-PCR. Nested-PCR primer sets to amplify exon 8–11 region were indicated as arrows (blue). NBD: nucleotide-binding domain, R: regulatory domain, TMD: transmembrane domain. **b**, **c** RT-PCR products of exon 8–11 in a patient J28 and his parents (**b**) and in J15 (**c**) in the nasal epithelial cells. Fa: father of J28, J28: patient, Mo: mother of J28, N: negative control. The nested-PCR primer 2F and 2R gave ~500 bp fragments in the father and 300 bp fragments in the patient and mother. **d** Confirmation of the RT-PCR products of exon 8–11 by Sanger sequencing. The sequence revealed exon 10 skipping in J28 and his father (upper), and partial exon 10 skipping in J15 (arrow)

The *CFTR* gene expression was investigated by reverse-transcription quantitative PCR (RT-qPCR)^20^. The primers and predicted sizes of the segments are shown in Fig. 1 and Supplementary Table S3. The glyceraldehyde-3-phosphate dehydrogenase (*GAPDH*) gene was amplified and considered as control. Each PCR product was run through agarose gel electrophoresis and then validated by the Sanger sequence.

### Statistical analysis

The variant frequencies in the Japanese population (except for splicing variants of intron 6 and 9) were obtained from in-house whole-exome sequencing data of 1500 non-pancreatitis controls or the Tohoku Medical Megabank Organization (ToMMo) 3.5k version 2 in the iJGVD^21^. The significance of the differences in variant frequencies between patients and controls was tested by two-tailed Fisher’s exact test, likelihood ratio test or *χ*^2^-test. A *p*-value of <0.05 was considered significant.

## Results

### Genetic analysis

Sequence analysis of the entire *CFTR* gene including promoter region, spanning about 200 kb, was performed in 28 patients by NGS. The 20 most common *CFTR* mutations found in Caucasian populations were not found in these patients (E60X, R117H, R334W, R347P, A455E, ∆I507, ∆F508, G542X, G551D, R553X, c.489 + 1G > T (previously noted as 621 + 1G > T), c.948delT (previously 1078delT), R1162X, S1251N, W1282X, N1303K, c.1585-1G > A (previously 1717-1G > A), c.2051_2052del insG (previously 2183AA > G), c.3528delC (previously 3659delC) and c.3718–2477C > T (previously 3849 + 10kbC > T)).

We identified 8 non-synonymous variants, R31C, E217G, R352Q, V470M, I556V, L1156F, Q1352H and R1453W, and 1 synonymous variant, c.2562T > G in 19 patients (Table 1). Of these variants, five are known to be unique to the Asian patients (E217G, I556V, L1156F, Q1352H, and R1453W)^12,14,16^. Of the eight non-synonymous variants, R31C was recorded as uncertain significance in the most severe clinical significance or conflict in the latest status in the ClinVar database. V470M was recorded as likely benign in both statuses in the ClinVar database. Others were recorded as pathogenic/likely pathogenic or conflict, respectively (Table 1). Allele frequency of R352Q and R1453W were significantly higher in patients compared with one in the in-house control data (*p* = 0.018 and 0.033, respectively, Table 1) and in the ToMMo_3.5K control population (*p* = 0.0078 and 0.044, respectively, Table 1)^21^. The R352 variant is known as pathogenic and R1453W is known as pathogenic in most severe significance but as conflict in the ClinVar database. Of four patients with CFTR R1453W variant, one patient had a variant of p.A137G in CPA1, which was previously reported^17^ (data not shown). In this study, we could not find novel pathogenic variants in exonic region of *CFTR* in the patients.

**Table 1:** Lists of *CFTR* variants detected in this study

Ch7 position (GRCh38)	Variant	dbSNP ID	Patient	Allele frequency Patient (%)	Allele frequency in house_1.5K (%)	*p*-Value	Allele frequency ToMMo_3.5K (%)	*p*-Value	CFTR mutation class^a^	Clinical significance in ClinVar	
										latest^b^	most severe^c^
A. Non-synonymous variants and a synonymous variant											
Promoter (117479129)	c.-966T > G/T c.-966T > G	rs4148682	11/28 4/28^d^	19/56 (33.9)	–	–	3075/7104 (43.3)	0.177	–	ND	ND
5′-UTR (117480087)	c.-8G > C/G	rs1800501	5/28	5/56 (8.93)	172/3000 (5.72)	0.222	366/7034 (5.20)	0.216	–	Conflict	Likely benign
ex2 (117504290)	c.91C > T/C p.R31C	rs1800073	1/28	1/56 (1.76)	24/3000 (0.8)	0.371	53/7092 (0.75)	0.347	II, III	Conflict	Uncertain significance
ex6 (117535318)	c.650A > G/A p.E217G	rs121909046	2/28	2/56 (3.57)	31/3000 (1.03)	0.122	74/7108 (1.04)	0.119	III, V	Conflict	Pathogenic
ex8 (117540285)	c.1055G > A/G p.R352Q	rs121908753	1/28	1/56 (1.76)	0/3000 (0)	0.018*	0/7104 (0)	0.0078*	III	Pathogenic	Pathogenic
ex11 (117587820)	c.1408G > A/G c.1408G > A p.V470M	rs213950	13/28 1/28^a^	15/56 (26.8)	1118/3000 (37.26)	0.067	2748/7106 (38.7)	0.074	III	Likely benign	Likely benign
ex12 (117587820)	c.1666A > G/A p.I556V	rs75789129	2/28	2/56 (3.57)	84/3000 (2.81)	0.472	235/7108 (3.32)	0.709	III	Conflict	Pathogenic
ex15 (117595001)	c.2562 T > G/T c.2562T > G p.T854=	rs1042077	13/28 1/28^d^	15/56 (26.8)	1113/1887 (37.1)	0.072	2742/7080 (38.7)	0.074	–	Likely benign	Likely benign
ex21 (117614713)	c.3468G > T/G p.L1156F	rs139729994	2/28	2/56 (3.57)	81/3000 (2.71)	0.453	204/7104 (2.87)	0.676	NA	Conflict	Likely pathogenic
ex25 (117664780)	c.4056G > C/G p.Q1352H	rs113857788	2/28	2/56 (3.57)	82/3000 (2.74)	0.459	168/7104 (2.36)	0.385	III, V	Conflict	Pathogenic
ex27 (117667022)	c.4357C > T/C p.R1453W	rs4148725	4/28	4/56 (7.14)	63/3000 (2.1)	0.033*	167/7102 (2.35)	0.044*	III	Conflict	Pathogenic

B. Splicing affecting variants											
Allele			Frequency						Fisher’s exact test		
c.1210-12T(5_9)			Patient (%)			Control (%)			*p*-Value		
5T			4/56 (7.14)			6/184 (3.26)			0.249		
6T			0			2/184 (1.09)			1		
7T			52/56 (92.9)			174/184 (94.6)			0.744		
9T			0			2/184 (1.09)			1		

C. Splicing affecting variants											
Genotype			Frequency						LR test		
c.1210-12 (splic, c.1210-34TG(9_13)			Patient (%)			Control (%)			*p*-Value		
7,11/7,11			8/28 (28.6)			22/92 (23.9)			0.622		
5,12/7,11			3/28 (10.7)			2/92 (2.17)			0.072		
6,12/7,11			0			1/92 (1.09)			0.111		
7,12/7,11			16/28 (57.1)			40/92 (43.5)			0.205		
7,12/9,11			0			1/92 (1.09)			0.111		
5,13/7,11			1/28 (3.57)			0			0.026*		
7,13/7,11			0			1/92 (1.09)			0.111		
7,13/9,11			0			1/92 (1.09)			0.111		
5,12/7,12			0			3/92 (3.26)			0.057		
6,12/7,12			0			1/92 (1.09)			0.111		
7,12/7,12			1/28 (3.57)			18/92 (19.6)			0.022*		
5,13/7,12			0			1/92 (1.09)			0.111		
7,13/7,12			0			1/92 (1.09)			0.111		

*NA* not applicable, *ND* no data

^a^Evaluated by Vankeerberghen et al.^34^

^b^Latest (on 15th February 2019)

^c^Most severe clinical significance, *p  < 0.05

^d^Homozygote

A splice-affecting variant, 5_9T (c.1210-12 T(5_9)), in intron 9 was investigated in the patients and controls by Sanger sequencing (Table 1b). The heterozygous variant 5T was identified in four patients, with an allele frequency of 7.14% (Table 1b). The allele frequency of 5T was not significantly different (*p* = 0.249) between patients and controls (Table 1b).

Many intronic variants were found in patients (Supplementary Tables S4 and S5) and some of these variants, such as rs370483286 (intron 1), rs371779267 (intron 3), rs547233512 (intron 6), rs138454021 (intron 10), rs180877927 (intron 11), rs112433140 (intron 15), rs139568843 (intron 17), rs183819332 (intron 18), rs147410641 (intron 20), rs7797932 (intron 21), rs1820871 (intron 10), rs4148706 (intron 10), rs143964990 (intron 10), rs34855237 (intron 10), rs869218449 (intron 11), rs535033297 (intron 18), rs213985 (intron 21) and rs371815480 (3′-untranslated region), were significant (*p* < 0.05 and *p* < 0.01, respectively) in the patients compared with Japanese ToMMo control population^21^ and 1000 Genome data of the East Asian population (Supplementary Table S4)^22^.

Genotype of TG repeats 11/13 (c.1210-34TG (11/13)), which repeats expansion might also affect splicing^23^, and 5T/7T (c.1210-12 T (5/7)), both in intron 9, was found in 1 patient but not in 92 normal controls (Table 1C and Table 2 (J28)).

**Table 2:** Variants of the *CFTR* gene and clinical feature of patients

Patient	*CFTR* genotypes				Clinical feature					
	Non-synonymous		Synonymous	c.1210-12 T(5_9), c.1210-34TG(9_13)	Sex	Onset age	Main symptoms	Structural abnormality	Sweat chloride concentration	Family history
J1	**L1156F** **<** ND >	V470M	c.2562 T>G/T	7,11 / 7,12	M	1	ND	−	Normal	ND
J3	**E217G** < ND >	V470M	c.2562 T>G	7,12 / 7,12	F	2	Drug-induced	−	Normal	ND
J4	**R1453W** < Mo >	V470M	c.2562 T>G/T	7,11 / 7,12	M	2	Abdominal pain	−	Normal	+
J5	**R352Q** < Mo >	V470M	c.2562 T>G/T	7,11 / 7,12	F	4	Abdominal pain	−	Normal	−
J6	**Q1352H** < Fa>			7,11 / 7,11	M	4	Abdominal pain	+	Normal	+
J8	**L1156F** **<** Mo >	V470M	c.2562 T>G/T	7,11 / 7,12	ND	UC	ND	ND	ND	ND
J12	**I556V** < Fa>			7,11 / 7,12	F	UC	ND	ND	ND	ND
J15	**R1453W** < Mo>	R31C < Fa>		7,11 / 7,11	F	2	ND	+	Normal	−
J16	**I556V** < Mo>			7,11 / 7,12	F	6	ND	ND	ND	ND
J18	**R1453W** **<** Mo >	V470M	c.2562 T>G/T	7,11 / 7,12	M	6	ND	ND	ND	ND
J19	**R1453W** < Mo >	V470M	c.2562 T>G/T	7,11 / 7,12	M	4	Abdominal pain	−	Normal	+
J21	**E217G** < ND >	V470M	c.2562 T>G/T	7,11 / 7,12	F	11	Abdominal pain	+	ND	−
J23	**Q1352H** **<** Mo >			7,11 / **5**,12	F	13	Abdominal pain	+	Normal	+
J26		V470M	c.2562 T>G/T	7,11 / **5**,12	F	11	Abdominal pain	−	Normal	ND
J27				7,11 / **5**,12	M	UC	ND	−	ND	+
J28				7,11 / **5**,13	M	8	Abdominal pain	−	Normal	+

Pathogenic or associated variants are indicated in bold

*< >* genotype origin, *Fa* father, *Mo* mother, *ND* no data, *UC* uncertain

For pathogenic variants, seven non-synonymous variants and one splice-affecting variant in intron 9 of *CFTR*, patients with variants and clinical feature were listed in Table 2 (five patients with V470M only are not listed). Of the 28 patients in this study, 16 patients (57.1%) had non-synonymous or splice-affected variant of *CFTR* (Table 2). There was no effect of sex on these findings. Onset of the disease ranged from 1 to 13 years. The main symptom of the patients was abdominal pain. The sweat chloride concentration test of patients, who had a result, was normal. There does not seem to be an obvious correlation between variant type and age of onset. In this study, patients with abnormal findings of endoscopic retrograde cholangiopancreatography and/or magnetic resonance cholangiopancreatography, such as stones and pancreatic duct dilatation, had a non-synonymous variant of R31C, E217G or Q1352H, which has been previously reported in chronic pancreatitis in adult patients^14,16,24^ (Table 2).

### RNA analysis

Patient J28 had a genotype of (TG) 11/13, (T) 5/7 in intron 9, which might affect splicing of *CFTR* (Table 2). One allele of (TG)13, (T)5 was inherited from his mother, who was affected with alcoholic-related pancreatitis. Results of RT-PCR for exon 8–11 of *CFTR* displayed a shorter fragment compared with control in the patient and the mother, but not in the father (Fig. 1b). Sanger sequencing of the RT-PCR product from the family revealed that exon 10 of *CFTR* was skipped in the nasal epithelial cells (Fig. 1d).

We analysed the *CFTR* gene expression in two patients, J15 and J28, with RT-qPCR^20^ and RT-PCR. J15 had a compound heterozygote of R1453W and R31C inherited from non-affected mother and father, respectively. *CFTR* expression was investigated in the nasal epithelial cells by RT-qPCR using primer pairs targeted for two regions (Fig. 1a; I and II). Gene expression and splicing pattern were not altered compared with normal control (data not shown).

We also analysed the splicing of *CFTR* in J15, who had genotype of (TG) 11/11, (T) 7/7 in intron 9 (Table 2). Partial skipping of exon 10 was observed in the patient (Fig. 1c, d).

## Discussion

In this study, we investigated the entire *CFTR* gene in 28 Japanese paediatric patients with idiopathic pancreatitis using targeted NGS analysis and *CFTR* expression analysis. We found that 16 patients had non-synonymous or splice-affecting variant of *CFTR* out of 28 patients, who had no pathogenic variants of *PRSS1* and *SPINK1* by genetic analysis in our previous study^17^. We found eight non-synonymous variants of *CFTR*, R31C, E217G, R352Q, V470M, I556V, L1156F, Q1352H and R1453W, in the patients. R352Q was recorded as pathogenic in the ClinVar database (Table 1). The other variants, except V470M, are registered as conflict pathogenicity in the Clinvar database (Table 1). The pathogenicity of such variants was registered as based on CF-causing variants. In fact, the variants found in this study are known to affect function or expression of CFTR as described later, suggesting that those are functionally affected variants.

Up to now, *CFTR* analysis has been neglected in Japan as *CFTR* variants are thought to be rare in Japanese CF patients^25,26^. However, our findings imply that Japanese paediatric patients with idiopathic pancreatitis would have greater rates of *CFTR* variants than previously expected.

No patients in our study had any of the common CF-causing variants in Caucasians, consistent with other studies in Asian patients^12–16,24^. This suggests that Asian or Japanese populations may have different variants of *CFTR* than European populations. Of the eight non-synonymous variants found in the patients, it appears that two variants, L1156F and R1453W, are unique to Japanese patients^12–16,24^ and Q1352H is known to be unique in Asian patients^13,14,16,24^. These reports were all in the Japanese or Asian samples, and data from 1000 Genomes studies suggest no variants are found in other ethnicities.

In this study, eight non-synonymous variants, R31C, E217G, R352Q, V470M, I556V, L1156F, Q1352H and R1453W, were found in our patients. Nine out of 28 patients had a V470M variant (Table 2). It is known that channel activity of V470 CFTR protein is lower than that of M470 CFTR^23,24^. Cuppens et al.^23^ suggested that M470 CFTR proteins matured more slowly than V470 CFTR, although M470 CFTR had higher channel activity than V470 CFTR. Hence, the proteins perform complementary functions to each other, suggesting that the variant is benign. However, these studies displayed that the V470M locus affected the penetrance of another variants’ expression, such as c.1210-12 T(5)^14,24^. There are many reports about the contribution of the V470M locus to other variants^13–16,23,24^ and it is possible that the V470M locus affects the penetrance of other functionally affected variants, such as E217G, R352Q, L1156F, and R1453W. However, more experiments are needed to confirm these findings.

For the gene variants R31C, E217G, R352Q, I556V, L1156F, Q1352H and R1453W, there are reports that they affect protein activity and/or expression^14,24,27^. Lee et al.^14^ reported that the variants, E217G, I556V, Q1352H and R1453W of CFTR decreased channel activity, which implies an association with pancreatitis in Japanese patients. The L1156F CFTR, which is associated with alcoholic chronic pancreatitis in the Japanese, causes impaired CFTR function and expression in combination with the V470M variant^24^. This variant might also affect paediatric pancreatitis.

**Fig. 2:**
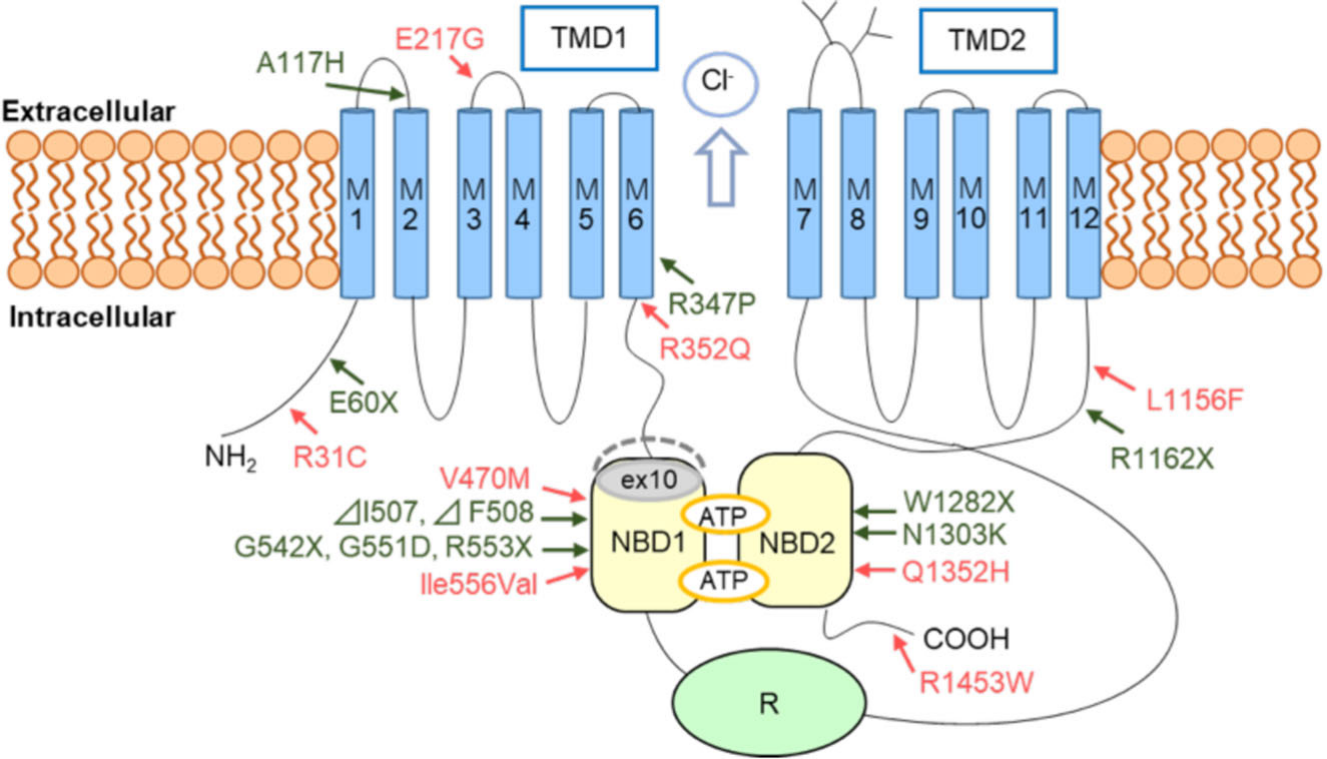
Schematic diagram of the predicted protein structure of CFTR and locations of variants. The non-synonymous variants, which were found in this study, indicated as arrows in red colour. The common non-synonymous variants in Caucasian were indicated as arrows in green colour. The amino acids encoded in exon 10 (within the NBD1 domain) were indicated as shaded areas (grey)

The R352Q and R1453W variants were significantly more frequent in patients (Table 1). According to the Exome Aggregation Consortium, the R352Q variant is rare in Caucasian population with an incidence of 6 in 121,412 (0.0002%)^28^. The R352 CFTR is a residue flanking the predicted cytoplasmic end of the M6 segment (Fig. 2) and the Q352 leads to a decrease in anion-selective activity in the channel^29^. The R1453W variant is not found in either Chinese and Korean patients with idiopathic pancreatitis^12,14^. The W1453 CFTR variant also affects the protein function and gene expression^14^. In the previous study, two patients out of 128 patients had a heterozygous variant of p.A137G in CPA1^17^. Of the two patients, one patient had also had the p.R1453W variant in CFTR, suggesting that the patient was affected not only by CPA1 A137G but also CFTR R1453W.

We evaluated the *CFTR* gene expression in patient J15, who had two heterozygote variants, R31C and R1453W. Jurkuvenaite et al.^27^ reported that R31C leads to decreased expression at the cell surface and diminished Cl^−^ channel activity. The expression analysis showed that neither variant affected *CFTR* gene expression and splicing in our patient. Lee et al.^14^ reported that R1453W-CFTR showed mild reduction of open probability. Thus, both variants might be associated with the development of pancreatitis after translation.

A splice-affecting variant, 5T in intron 9 was identified in four patients. We detected exon 10 skipping of *CFTR* in J28 and his mother in the nasal swab cells (Fig. 1). Both had long TG repeats (13TG) adjacent to 5T, which confirms the previous study results that longer TG repeats increase risk of exon 10 skipping^30^. The transcripts completely skipped exon 10, whereas the patient had 11TG repeat and 7T in another allele. Rave-Harel et al.^31^ reported that the degree of exon 10 skipping with variant 5T was variable and penetrance of disease expression was also different in individuals. Although patient J15 and the non-affected father of J28 have the same genotype, (TG) 11/11 and (T) 7/7, partial skipping of CFTR exon 10 occurred only in patient J15, suggesting that other genetic factors might affect splicing^31^.

The skipping of exon 10 causes the deletion of first 21% of nucleotide-binding domain 1 where the common CF mutation ∆508 is found in Caucasians^5,32^. The domain seems to be a critical region associated to diseases. Patient J28 and his mother had no symptoms of respiratory involvement and only of pancreatitis. This suggests that exon 10 skipping might affect pancreatitis more so rather CF.

We found many intronic variants in the patients with paediatric pancreatitis, which were unique or significantly more frequent compared with the Japanese population (Supplementary Tables S4, 5). According to the association study, it was reported that some synonymous and/or non-exonic variants are involved in pancreatitis^24^. Therefore, it is possible that some variants are involved in genetic risk factors for paediatric pancreatitis. However, further experiments should be needed to evaluate the relationships between intronic variants and pancreatitis.

In the present study, the frequency of pathogenic and functionally affected *CFTR* variants in Japanese paediatric patients with pancreatitis was 1/28 (3.6%) and 15/28 (53.6%), respectively. However, as patients in this study were not found to have pathogenic variants in the causative genes for pancreatitis in a previous study, the actual frequency in pancreatitis is likely lower. Taken together with our previous study^17^, the frequency of having such pathogenic or related variant of *CFTR* is at least more than 12.5% (16/128). As the *CFTR* gene is not causative but rather an associate gene for pancreatitis, pathogenic variants may affect developing pancreatitis^33^. The *CFTR* pathogenic or functionally affected variants found in this study may affect pancreatitis in Japanese populations as well.

In summary, we found that one out of 28 (3.6%) and 15 out of 28 (53.6%) Japanese patients with paediatric pancreatitis had a pathogenic and functionally affected variant in *CFTR*, respectively. As the 28 patients studied were not diagnosed after genetic analysis of known causative genes for pancreatitis in a previous study^17^, the actual frequency of *CFTR* variants in Japanese paediatric pancreatitis will be lower than demonstrated in this study. Considering results of the present and previous studies, this suggests that the frequency of functionally affected variants of *CFTR* is estimated to be at least 12.5%. Variants of the *CFTR* gene in the Japanese were previously thought to be rare, as CF is a very rare disease estimated at 1/350,000 in Japan^25^. However, this study suggests that pathogenic or functionally affected variants of *CFTR* might not be so rare in Japanese paediatric patients with idiopathic pancreatitis. Thus, *CFTR* may also present as a genetic risk factor in paediatric pancreatitis in Japanese. Surveying variants of the *CFTR* gene might help determine risk of pancreatitis in Japanese children.

## Supplementary information

Primers and PCR conditions for LA-PCR of the CFTR gene

Primers for Sanger sequencing of the CFTR gene

Primers for expression analysis (RT-PCR) of CFTR

CFTR intronic and UTR variants found in the patients with idiopathic pancreatitis in this study

List of CFTR intronic and UTR variants in the patients
