# Supplementary material for: The CFTR gene variants in Japanese children with idiopathic pancreatitis
Source: Hum Genome Var. 2019 Apr 11;6:17. doi: 10.1038/s41439-019-0049-7 (PMC6459923; doi:10.1038/s41439-019-0049-7)
Supplement: Supplementary file 1 — Primers and PCR conditions for LA-PCR of the CFTR gene [file 41439_2019_49_MOESM1_ESM.docx]

Table S1. Primers and PCR conditions for LA-PCR of the *CFTR* gene
