# Supplementary material for: The CFTR gene variants in Japanese children with idiopathic pancreatitis
Source: Hum Genome Var. 2019 Apr 11;6:17. doi: 10.1038/s41439-019-0049-7 (PMC6459923; doi:10.1038/s41439-019-0049-7)
Supplement: Supplementary file 4 — CFTR intronic and UTR variants found in the patients with idiopathic pancreatitis in this study [file 41439_2019_49_MOESM4_ESM.docx]

Table S4. *CFTR* intronic and UTR variants found in the patients with idiopathic pancreatitis in this study

Allele frequencies are of ToMMo_3.5k_JPNv2 [21] and of 1000 GENOM_EAS [22].

* p < 0.05, ** p < 0.01　significant compared to ToMMo_3.5k_JPNv2.
