# Supplementary material for: The CFTR gene variants in Japanese children with idiopathic pancreatitis
Source: Hum Genome Var. 2019 Apr 11;6:17. doi: 10.1038/s41439-019-0049-7 (PMC6459923; doi:10.1038/s41439-019-0049-7)
Supplement: Supplementary file 5 — List of CFTR intronic and UTR variants in the patients [file 41439_2019_49_MOESM5_ESM.docx]

Table S5. List of *CFTR* intronic and UTR variants in the patients

* *p* < 0.05, ** *p* < 0.01　difference from ToMMo_3.5k_JPNv2 [21].
